# miRNA in Endometriosis—A New Hope or an Illusion?

**DOI:** 10.3390/jcm14144849

**Published:** 2025-07-08

**Authors:** Anna Dryja-Brodowska, Bogdan Obrzut, Maciej Obrzut, Dorota Darmochwał-Kolarz

**Affiliations:** 1 Department of Obstetrics & Gynecology, Faculty of Medicine, University of Rzeszow, Rejtana 16C, 35-959 Rzeszow, Poland; 2 Faculty of Medicine, University of Zielona Góra, Licealna Str 9, 65-417 Zielona Góra, Poland

**Keywords:** microRNA, miRNA, endometriosis, non-invasive biomarkers, gene expression, reproductive health

## Abstract

**Background:** Endometriosis is a complex, estrogen-dependent condition that can significantly impact women’s quality of life and fertility. Current diagnostic strategies remain invasive and often prolonged, demonstrating the need for reliable, non-invasive biomarkers. In this context, microRNAs (miRNAs), due to their stability in blood and regulatory roles in inflammation and cell proliferation, have emerged as promising candidates. **Methods:** This review systematically analyzes 17 studies published between 2010 and 2025 that investigated the diagnostic utility of circulating and tissue-based miRNAs in endometriosis. **Results:** A wide range of dysregulated miRNAs was identified, with miR-125b-5p, miR-451a, and miR-3613-5p showing the most consistent alterations across studies. However, diagnostic performance varied considerably—largely due to methodological heterogeneity. Key differences were observed in sample type (serum, plasma, endometrium), patient selection, and control group definition. The menstrual cycle phase and hormonal status were often not matched or reported, limiting reproducibility. **Conclusions:** Despite encouraging findings, the current evidence base is weakened by inconsistent protocols and limited validation. Standardized, multicenter research with well-characterized patient cohorts is essential to the establishment of clinically applicable miRNA-based diagnostics. If validated, miRNAs may offer a transformative, non-invasive approach for earlier detection and improved management of endometriosis.

## 1. Introduction

Endometriosis is a chronic, estrogen-dependent inflammatory disease in which endometrium-like tissue develops outside its natural location—within the ovaries, fallopian tubes and other pelvic structures, and—in rare cases—in other parts of the body [1].

According to the World Health Organization (WHO), endometriosis affects 6–13% of the reproductive age population [2]. Some sources indicate that the incidence in the general population may be as high as 15–18% [3,4] and, in women struggling with infertility, this percentage can be as high as 30–50% [5]. There are also reports of isolated cases of endometriosis in men [6].

Pain is the dominant symptom of this disease [7,8,9]. It is not only a physical symptom but also a factor influencing the emotional and social life of patients [10]. Its chronicity and variability can lead to serious consequences, in terms of both physical and mental health—including depression, weakening of relationships with loved ones and problems with self-esteem [11,12]. Women suffering from endometriosis may avoid professional, educational, social, or family activities, that have further consequences in the form of reduced economic activity and a decrease in the number of people participating in the workforce [13,14,15].

Endometriosis is a disease that is often difficult to diagnose due to its varied symptoms, which may resemble other diseases such as primary dysmenorrhea, pelvic inflammatory disease, intestinal problems, urinary tract inflammation, or degenerative changes in the spine, which presents a diagnostic challenge for doctors of all specialties [16]. It is a difficult to diagnose disease due to the lack of non-invasive tests that would allow its detection [17].

Until recently, laparoscopy was considered the gold standard in diagnosing endometriosis [18]; however, this is an invasive procedure and carries a risk of complications. Furthermore, the absence of endometriosis foci during surgery does not necessarily entail a negative diagnosis. The latest ESHRE guidelines recommend diagnosing endometriosis based on an interview and/or imaging tests, i.e., ultrasound or MRI [19]. According to the ESHRE guidelines, surgery should be proposed to patients with symptoms that do not respond to pharmacological treatment or with symptoms but with negative imaging test results.

Therefore, the search for a non-invasive marker enabling the diagnosis of endometriosis has become a priority for researchers in recent years [20].

Among the numerous non-invasive markers of endometriosis being studied are microRNA.

microRNAs (miRNAs) are short, endogenous RNA molecules of about 22 nucleotides in length that play a key role in the regulation of gene expression at the post-transcriptional level [21]. They act by binding to mRNA and inhibiting translation, leading to the suppression of target gene expression [22].

In addition, miRNAs can influence chromatin modifications and DNA methylation, which further regulates gene expression [23].

miRNAs are involved in many biological processes such as cell division, proliferation, cell differentiation, apoptosis and stress response [24]. Because of their high stability in biological fluids and tissue specificity, miRNAs could be promising candidates for diagnosing endometriosis and other diseases [25,26].

In recent years, researchers have shown that specific miRNAs are important in endometriosis [27].

In this article, we aim to assess whether we are indeed approaching the discovery of a new, non-invasive biomarker for endometriosis, as suggested by the growing body of research, or whether this is merely an illusion resulting from the large number of papers on the subject.

We aim to explore the current state of knowledge on this subject by analyzing the available articles that may indicate a real breakthrough in the diagnosis of endometriosis and to identify possible barriers that may prevent full achievement of this goal.

## 2. Materials and Methods

### 2.1. Protocol

The protocol for this systematic review was designed following the guidelines of the Preferred Reporting Items for Systematic Review and Meta-analysis Protocols (PRISMA) [28]. The present review protocol is officially registered in the INPLASY database on 6 June 2025 (INPLASY202560027).

### 2.2. Search Strategy

We conducted a thorough literature review using electronic databases such as Pubmed and Google Scholar, considering articles that examined miRNA as potential biomarkers in endometriosis. Articles were published in English from 2010 to March 2025. We used keywords such as: micro RNA, miRNA, biomarker, endometriosis, mcRNA and a combination of them. We found 727 articles.

Inclusion criteria contained: randomized clinical trials, meta-analyses, retrospective and prospective studies; human studies only; publication year between 2010 and 2025; articles in English; accessible as full-text articles; study population ≥ 24; samples taken from blood, serum, plasma, or endometrium; journal Impact Factor at the time of publication ≥ 2.

Exclusion criteria included: not accessible as full-text articles; articles in a language other than English; non-human study population; Impact Factor < 2; publication year earlier than 2010; study population < 24; review articles. The exclusion of studies published in journals with an impact factor (IF) below 2 was implemented to reduce the risk of including poorly controlled or non-peer-reviewed studies and to prevent inclusion of repetitive or lower-quality results from less rigorous publications. While this criterion may have omitted some relevant data, it was implemented to strengthen the overall credibility and scientific rigor of the review.

Full-text articles were obtained for abstracts that appeared to meet the inclusion criteria. We additionally conducted a manual review of the bibliographies of the included articles to identify additional relevant studies.

Taking into account the inclusion and exclusion criteria listed below, we selected 17 articles for this review.

### 2.3. Data Collection Process

The articles included in our review were thoroughly analyzed by two independent researchers. Authors performed data extraction using a Microsoft Excel form (Microsoft Corporation, Redmond, WA, USA). The following data were obtained from each study: the name of the first author, year of publication, the journal’s Impact Factor at the time of publication, the nationality of the patients included in the study, the number of participants in the study group and control groups, the phase of the menstrual cycle—whether it influenced miRNA expression, key dysregulated miRNA, control group characteristics, and the main outcomes.

A formal risk of bias assessment was not performed due to the heterogeneity of study designs included. However two independent reviewers evaluated each study for eligibility based on the predefined criteria outlined in the protocol. Any differences between the reviewing authors were clarified in joint discussions. Based on the 17 studies included in this review, several recurring sources of potential bias were identified. Selection bias was common, as control groups often included women with other gynecologic conditions rather than healthy, asymptomatic individuals. Many studies did not report or control for the menstrual cycle phase at the time of sample collection. Sample types (serum, plasma, tissue) and analytic platforms varied widely, limiting comparability. Small sample sizes and lack of independent validation cohorts further reduced the generalizability of findings. Such factors may have influenced the comparability and reproducibility of results across studies.

**Figure 1:**
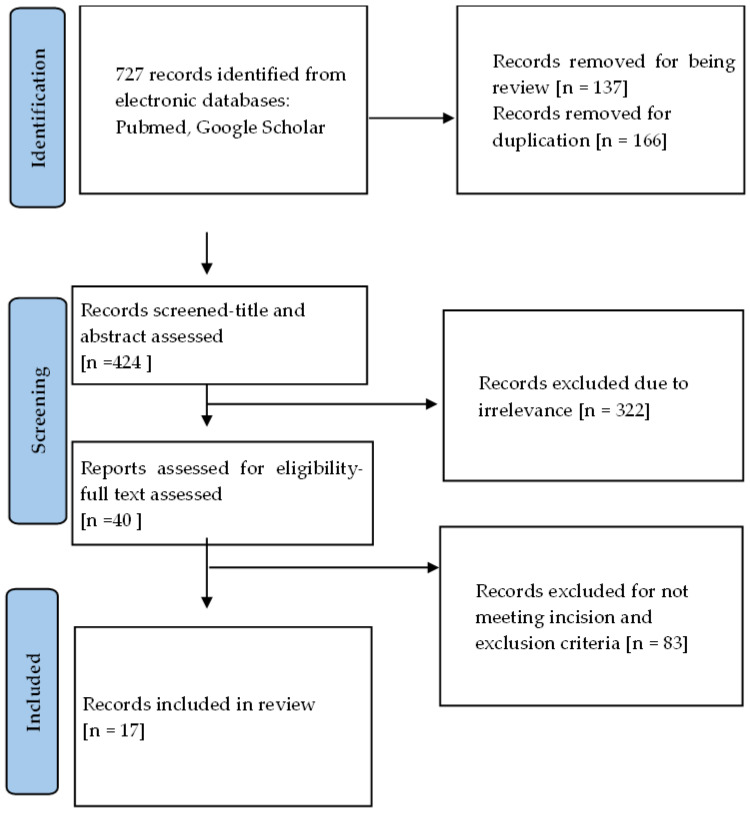
Flow diagram illustrating the search strategy and selection process in line with PRISMA guidelines.

The results of the identified studies were organized into a table, with the most significant elements discussed in detail. The study selection process is illustrated in the PRISMA flow diagram (Figure 1).

## 3. Results

This review included 17 studies examining miRNA in women with endometriosis compared to controls [29,30,31,32,33,34,35,36,37,38,39,40,41,42,43,44,45]. Despite the diversity of methods and study populations, common patterns and important differences in study designs can be identified.

Table 1 provides an overview of all included studies and their key characteristics such as the name of the first author, the year of publication, the country where study was performed, the journal’s Impact Factor at the time of publication, the number of patients included in study group and control group, whether menstrual phase was taken into account and its result on miRNA expression, dysregulated miRNA-upregulated or downregulated, sample type and characteristic of control group.

### 3.1. Sample Type

The vast majority of the studies included in this review (12 out of 17) used serum or plasma samples to evaluate miRNA levels as potential non-invasive biomarkers [30,32,33,34,36,37,38,39,40,41,42,43,45]. Serum was used in six studies [30,32,34,39,43,45]. The same number of studies used plasma [33,36,37,38,40,42]. Only one study [21] used white blood cells to investigate single nucleotide polymorphisms (SNPs) in miRNA genes, rather than miRNA expression itself.

Three out of 17 studies [29,31,35] focused on the analysis of eutopic and/or ectopic endometrial tissue and only one study [44] used only endometrial lesions for analysis.

### 3.2. Validation Method

RT-qPCR was used as a validation method in 14 out of the 17 studies [29,30,31,32,33,34,35,36,37,38,39,40,42,44]. The remaining three studies were designed differently [41,43,45]. Farismadan et al. analyzed genetic polymorphisms (SNPs) rather than expression levels [41]. Wang et al. (2025) used miRNA microarrays, but did not clearly describe the qPCR-based validation of individual miRNAs [45]. Chico-Sordo et al. used multiplex analysis and machine learning, but did not report qRT-PCR validation [43].

### 3.3. Number of Studies by Continent

A review of the included studies revealed a predominance of research conducted in Asia (n = 7) [30,32,35,40,41,44,45], particularly in countries such as China, Korea, Iran, and Iraq (Table 2). European countries accounted for six studies (Spain, Austria, Belgium, Poland, and Estonia) [31,33,36,38,42,43], while a smaller number of studies originated from North America (n = 3, all from the USA) [29,34,39] and Oceania (n = 1, Australia) [37].

### 3.4. Study and Control Group Characteristic

All women in the study groups from selected publications had endometriosis confirmed surgically and histopathologically. The reviewed studies showed inconsistent reporting of coexisting disorders.

Across the 17 studies, the characteristics of the control groups varied considerably.

In 12 out of 17 studies, the control participants underwent laparoscopic surgery because of pelvic pathology [29,32,33,34,36,37,38,39,40,41,42,44]. The most common indications in this subgroup included tubal abnormalities, benign adnexal masses, e.g., ovarian or paraovarian cysts, uterine fibroids or pelvic pain.

Besides the above-listed pelvic pathologies, infertility as a co-indication was also mentioned in six papers [30,35,36,37,38,40].

In two studies, controls underwent laparoscopy specifically for sterilization [31,43].

The study of Farismadan et al. included controls undergoing surgery for CIN III (cervical intraepithelial neoplasia); however, the type of surgical procedure was not specified in the study [41]. In the research of Petracco et al., the authors did not specify whether the women in the control group underwent surgery [29].

Notably, only one study [45] involved a control group consisting of healthy, non-operated women who were asymptomatic and had normal levels of reproductive hormones.

### 3.5. Phase of Menstrual Cycle

**Figure 2:**
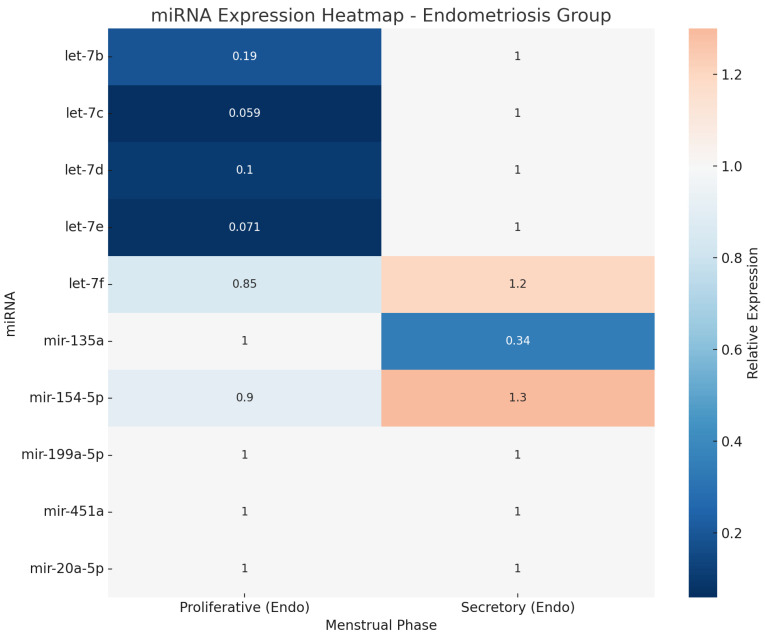
Diagram (heatmap) illustrating miRNA expression in endometriosis group depend on menstrual phase.

**Figure 3:**
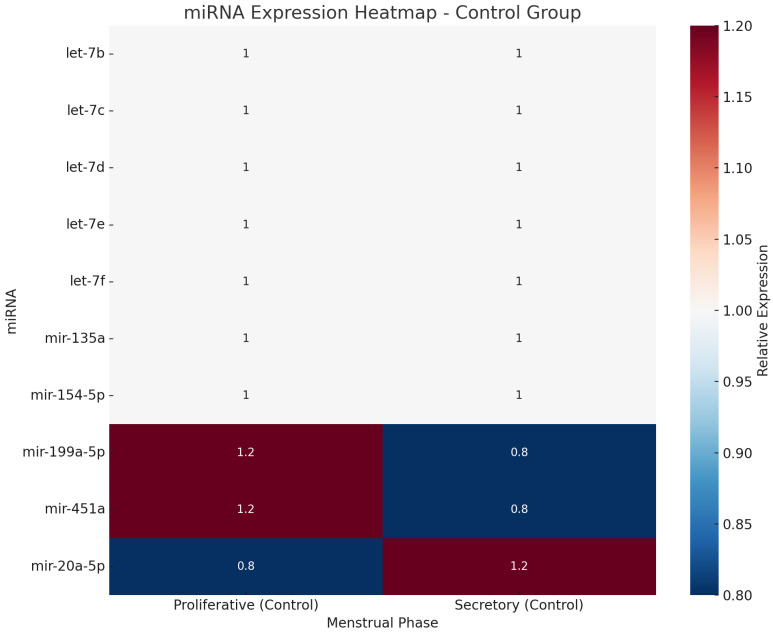
Diagram (heatmap) illustrating miRNA expression in the control group depends on the menstrual phase.

The menstrual cycle phase was considered in 10 out of the 17 included studies [29,30,31,32,33,34,36,37,38,39]. However, only three of these studies demonstrated a significant influence of menstrual phase on miRNA expression [32,36,37]. The phase-dependent miRNAs identified in these three studies have been visualized in two separate diagrams (Figure 2 and Figure 3), presented as heatmaps, to illustrate differential expression patterns across menstrual phases in women with endometriosis and healthy controls.

One study considered the cycle phase only in the control group [43].

In one study, all participants in the endometriosis group were in the mid-secretory phase [35].

Of the 17 studies, 5 do not take into account the menstrual cycle phase [40,41,42,44,45].

### 3.6. Dysregulated miRNA Across Studies

Analysis of dysregulated miRNAs revealed a set of molecules commonly identified in multiple studies. Among these, miR-125b-5p was reported upregulated in three studies [34,38,39], as were miR-451a [34,39,42] and miR-3613-5p was downregulated [34,39,42].

Other recurrent miRNAs included miR-135a upregulated [29,32], let-7b downregulated [32,39]; miR-150-5p upregulated [34,39]; miR-342-3p upregulated [34,39] and miR-143-3p downregulated [34,40].

Analysis of dysregulated miRNAs by sample type revealed that miRNA was both upregulated and downregulated in serum as well as in plasma [30,32,33,34,36,37,38,39,40,41,42,43,45].

In contrast, in all studies analyzing endometrial or endometriotic tissue, all miRNAs investigated were upregulated [29,31,35,44].

### 3.7. Impact Factor of the Studies

The impact factor (IF) of the journals in which the analyzed studies were published ranged from 2.3 to 8.8. The median IF was 4.8. This distribution indicates that this area is supported by a reliable source of peer-reviewed scientific publications.

## 4. Discussion

miRNAs play a key role in the pathogenesis of endometriosis, influencing many biological and molecular processes involved in the development and persistence of endometrial lesions outside the uterine cavity [46,47]. They contribute to the development of endometriosis by modulating processes such as inflammation, cellular proliferation and apoptosis, angiogenesis, extracellular matrix remodeling, tissue repair and TGFβ-regulated pathways, hypoxic injury and resistance to progesterone [48,49,50].

Numerous genes involved in endometriosis are regulated by miRNAs. Among the most prominent are ERα/β, KRAS4A/4B, CYP19, IL-6, HOXA9, HOXA10, and EDN1—all relevant to disease pathophysiology. miRNAs further modulate mTOR signaling and the VEGF pathway, affecting cell growth, migration, and angiogenesis—key mechanisms in lesion development [51,52,53,54].

In recent years, miRNAs have gained enormous interest as potential non-invasive diagnostic biomarkers in endometriosis.

Efforts to develop non-invasive diagnostic tools for endometriosis continue to intensify, reflecting the condition’s widespread nature and its profound clinical and societal consequences [20]. In the United States alone, the annual economic burden of endometriosis has been estimated at $78 billion, factoring in healthcare costs and productivity losses [15]. A large multicenter study involving over 1400 women demonstrated that patients with endometriosis worked, on average, 10.8 h less per week compared to controls, primarily due to pain-related impairment [13].

Moreover, chronic inflammation and persistent immune activation associated with the disease may contribute to increased risk of comorbidities, including cardiovascular disease, autoimmune disorders, and endocrine dysfunctions [55,56].

These associations further emphasize the urgency to identify effective, non-invasive diagnostic strategies that could facilitate earlier detection and targeted interventions.

Several systematic reviews have examined circulating miRNAs in the context of endometriosis diagnosis, yet they consistently report inconsistent findings and a lack of validation in independent cohorts. Agrawal et al. [57] identified 42 dysregulated miRNAs across nine studies, but only one miRNA (miR-20a) was consistently reported, underscoring the limited reproducibility of candidate markers. Similarly, Leonova et al. [58] found that out of 63 miRNAs reported in 18 studies, only 14 were replicated, attributing these discrepancies to methodological variability. Earlier reviews, such as the Cochrane analysis by Nisenblat et al. (2016) [59], evaluated a broad range of blood biomarkers but gave minimal attention to miRNAs and did not assess their diagnostic value in depth.

Our systematic review offers a focused and up-to-date synthesis of recent miRNA studies, critically evaluates their methodological quality, and proposes structured recommendations to enhance their diagnostic use.

This review of 17 studies reveals both the great potential of miRNA as new non-invasive biomarker for endometriosis and significant methodological and interpretive limitations related to their clinical application. These challenges must be critically examined before any meaningful clinical proceeding can be proposed.

One of the main limitations of this review, as with any comprehensive analysis, is the substantial methodological variability across studies—including differences in sample materials, experimental models, analytical techniques, and outcome measures—which often leads to conflicting results and complicates the interpretation of the overall evidence.

A recurring issue across the literature is the limited size of study cohorts. Many investigations included small, often non-representative groups, both in the study and control group. Such sample sizes undermine statistical reliability and make it difficult to generalize findings. Additionally, subgroup analyses based on disease stage, lesion localization, or symptom severity are typically underpowered or absent altogether, leading to oversimplified conclusions.

Compounding this issue is the heterogeneity of control groups used across studies. Rather than recruiting healthy, asymptomatic women, many researchers have included patients undergoing surgery for other gynecological conditions such as fibroids, cysts, or unexplained infertility. These comorbidities may independently influence miRNA expression profiles, confounding comparisons and obscuring disease-specific signatures. Numerous studies have demonstrated that miRNAs are dysregulated in uterine fibroids [60,61,62]. In asymptomatic women undergoing sterilization, studies have shown that the incidence of endometriosis varies between 2 and 43% [63,64]. In women with unexplained infertility, the incidence of endometriosis may range from 30% to 63.2% [65]. Among women hospitalized for chronic pelvic pain, endometriosis is diagnosed in 45% to 82% of cases [66]. Furthermore, inflammatory or hormonal abnormalities present in these conditions may mimic or mask endometriosis-related miRNA changes. On the other hand, it is important to recognize that laparoscopy, currently considered the gold standard for confirming endometriosis, remains an invasive procedure associated with operative risks and ethical concerns. As a result, designing a truly “healthy” control group, that can be definitively confirmed as endometriosis-free without surgical exploration, presents a significant methodological challenge. Identifying appropriate control participants is further complicated by the fact that endometriosis may be asymptomatic [17]. Enrolling women without any gynecological complaints and excluding laparoscopy for ethical reasons may lead to undetected cases within control cohorts. This limitation highlights the delicate balance between diagnostic accuracy and ethical feasibility in the selection of appropriate control populations for endometriosis research.

In many of the reviewed studies, the risk of incorrect classification of participants into endometriosis or control groups could not be excluded. Publications did not provide sufficient details concerning the diagnostic criteria, surgical techniques employed, and the qualifications or experience level of the surgical team.

A further confounder is the neglect of menstrual cycle phase. It is well documented that miRNA expression, particularly in endometrial and blood-derived samples, is modulated by hormonal fluctuations [67,68,69,70]. In the majority of studies, the menstrual cycle phase is either overlooked or insufficiently controlled, leading to biological variability. Controlling for or stratifying by cycle phase is essential, particularly when assessing tissues or fluids sensitive to estrogen and progesterone levels.

An additional limitation identified across the reviewed studies is the lack of geographic and ethnic diversity, with the majority of data derived from populations in Asia and Europe. Given that miRNA expression can be influenced by genetic, environmental and lifestyle factors [71], the inclusion of multi-ethnic cohorts is essential to ensure broader applicability and generalizability of findings.

A major obstacle in the field remains the inconsistency and poor reproducibility of results. Although several miRNAs have been identified as dysregulated in more than one study, especially miR-125b-5p, miR-451a, miR196 and let-7 family [32,34,35,36,38,39,41,42], the vast majority of reported miRNAs were unique to single studies [29,30,31,33,37,40,43,44,45]. This lack of replication limits the credibility of individual biomarkers and underscores the need for independent validation in diverse cohorts. This also suggests that miRNA profiles are highly context-dependent and susceptible to variations in methodology, sample handling, or population characteristics.

Another technical limitation relates to the lack of standardization in validation methods. Studies differ in their use of platforms such as RT-qPCR, microarray or NGS, normalization strategies, and analytical pipelines. Even when similar technologies are used, differences in RNA extraction, quality control, or thresholding can lead to divergent results. Without harmonized protocols, cross-study comparisons remain difficult, and findings lack the reliability needed for biomarker development.

In the article published by Rahmioglu et al. in 2014 [72], as part of the WERF EPHect (World Endometriosis Research Foundation Endometriosis Phenome and Biobanking Harmonization Project), the authors proposed recommended standard operating procedures (SOPs) for the collection, processing, and storage of biological fluid samples in endometriosis research. The aim of this international initiative, involving 34 clinical and academic centers across 16 countries, was to harmonize methodologies and enhance the comparability of findings across studies [72,73].

Taken together, these limitations highlight the urgent need for more rigorously designed, multicenter studies with larger sample sizes, homogeneous and well-defined control groups, standardized laboratory workflows, and thorough reporting of biological variables.

Only through such collaborative efforts can we advance toward identifying reliable, non-invasive biomarkers capable of aiding early and accurate diagnosis of endometriosis.

In light of the challenges described above, future research must shift from fragmented, exploratory studies toward collaborative, multicenter projects that follow standardized protocols and include larger, well-defined cohorts. Such studies should not only aim to replicate existing findings but also evaluate the diagnostic value of miRNA signatures in prospective, blinded settings.

Importantly, cycle phase stratification and documentation must become routine in all miRNA-related studies, especially when sampling endometrial tissue or blood. Likewise, consensus must be reached on best-practice laboratory techniques—from RNA isolation to quantification and data normalization—to enhance reproducibility and facilitate meta-analyses.

The goal should be to establish validated, reproducible biomarkers that complement current imaging and clinical assessment, thereby reducing diagnostic delay and guiding personalized treatment.

Until such rigorous efforts are undertaken, miRNAs should be considered as promising, but not yet ready-for-practice diagnostic tools. Their clinical translation will depend on overcoming the limitations discussed above and demonstrating consistent, replicable diagnostic performance across diverse populations.

## 5. Conclusions

miRNAs offer an exciting perspective in the context of developing non-invasive diagnostic biomarkers for endometriosis, but the current state of knowledge requires further research. Expression variability, methodological differences, lack of standardization, and lack of reproducible results in independent studies significantly limit their clinical value.

Before miRNAs find their place in everyday diagnostics, large, multicenter studies with a uniform protocol, standardization of data isolation, analysis, and reporting methods, consideration of biological and clinical variables, integration of miRNA data with other biomarkers (metabolomics, immune), and validation of candidates in prospective studies and ethnically diverse cohorts are necessary.

miRNAs remain a direction worthy of further research, but at present they are more of a scientific illusion than a clinical hope. Initial progress has been made, but considerable research is still required.

## 6. Future Directions

Several essential issues must be addressed to determine whether the growing interest in miRNAs as non-invasive biomarkers in endometriosis reflects a true clinical breakthrough. Firstly, the standardization of methodology. Existing studies are affected by methodological heterogeneity, including differences in RNA isolation protocols, normalization strategies, biofluid types, and timing of sample collection in relation to the menstrual cycle. Without standardized and reproducible approaches, comparison across studies remains limited. Harmonized protocols-ideally defined through international consensus—are essential for moving the field forward and for generating clinically reliable biomarker panels.

Another key area involves the integration of multi-omics approaches. miRNA expression patterns demonstrate promising diagnostic value, yet their accuracy, in terms of specificity and sensitivity, remains limited. Integrating miRNA levels with other molecular layers such as proteomics, metabolomics, or transcriptomics could greatly enhance diagnostic accuracy. Applying multi-omics techniques could uncover distinct molecular signatures underlying endometriosis, thereby improving disease stratification.

Finally, clinical translation remains a major challenge. To transition from exploratory findings to clinically applicable tests, large-scale prospective studies are urgently needed. These should include well-characterized patient cohorts, longitudinal sampling, and comparisons across disease stages and phenotypes. Additionally, regulatory approval (e.g., by the FDA or EMA) will require rigorous analytical and clinical validation of miRNA panels in diverse populations. A stepwise translational framework—moving from discovery to verification, validation, and commercialization—is currently lacking in the field.

## Figures and Tables

**Table 1:** Characteristics of studies included in the systematic review.

Author, Year	Country of Study	IF	Study Group (n)	Control Group (n)	Menstrual phase Considered (Yes/No)-Effect on miRNA Expression	Dysregulated miRNA	Sample Type	Characteristic of Control Group
Petracco et al., 2011 [29]	USA	6.3	32	50	yes-no effect	miR-135a ↑, miR-135b ↑	endometrial tissue	women undergoing operation-no data-women with polyps, uterine fibroids were excluded
Wang et al., 2013 [30]	China	6.3	60	25	yes-no effect	miR-199a ↑, miR-122 ↑, miR-145 ↓, miR-542-3p ↓	serum	women undergoing lpsc-mainly due to tubal infertility
Braza-Boïls et al., 2014 [31]	Spain	4.8	51	32	yes-no effect	miR-202-3p ↑, miR-424-5p ↑, miR-449b-3p ↑, miR-556-3p ↑	endometrial tissue, endometriotic lesions	women undergoing lpsc for sterilization
Cho et al., 2015 [32]	South Korea	4.4	24	24	yes-effect	let-7b ↓, let-7d ↓, let-7f ↓, miR-135a ↑	serum	women undergoing lpsc due to adnexal or peritubal cysts
Rekker et al., 2015 [33]	Estonia	4.4	61	65	yes-effect	miR-200a ↓, miR-200b ↓, miR-141 ↓	plasma	women undergoing lpsc with abdominal pain, infertility, PCOS or healthy-without lpsc
Cosar et al., 2016 [34]	USA	6.3	24	24	yes-no effect	miR-125b-5p ↑, miR-150-5p ↑, miR-342-3p ↑, miR-143-3p ↓, miR-451a ↑, miR-3613-5p ↓	serum	women undergoing lpsc due to adnexal or peritubal cysts
Zhou et al., 2016 [35]	China	4.6	22	20	yes, but only midsecretory-no data	miR-196a ↑	Endometrial tissue	women undergoing lpsc for infertility
Pateisky et al., 2018 [36]	Austria	2.9	51	41	yes-effect	miR-154-5p ↓, miR-196b-5p ↓, miR-378a-3p ↑, miR-33a-5p ↓	plasma	women undergoing lpsc with fibroids, infertility, adnexal cysts
Nisenblat et al., 2019 [37]	Australia	5.8	51	27	yes- effect	miR-155 ↓, miR-574-3p ↓, miR-139-3p ↓	plasma	women undergoing lpsc with abdominal pain and infertility
Vanhie et al., 2019 [38]	Belgium	5.7	60	30	yes-no effect	miR-125b-5p ↑, miR-28-5p ↑, miR-29a-3p ↑	plasma	women undergoing lpsc due to infertility, abdominal pain
Moustafa et al., 2020 [39]	USA	8.8	41	59	yes-no effect	miR-125b-5p ↑, miR-150-5p ↑, miR-342-3p ↑, miR-451a ↑, miR-3613-5p ↓, let-7b ↓	serum	women undergoing lpsc due to suspected benign pelvic lesions
Papari et al., 2020 [40]	Iran	7.3	53	53	no-no data	miR-199a-3p ↓, miR-143-3p ↓, miR-340-5p ↓, let-7b-5p, miR-21-5p ↑, miR-17-5p ↓, miR-20a-5p ↓, miR-103a-3p ↓	plasma	women undergoing lpsc with fibroids, infertility, pelvic tumors
Farismadan et al., 2021 [41]	Iran, Iraq	2.4	260	260	no-no data	miR-146a ↑, miR-149 ↑, miR-196a2 –, miR-499 (their SNPs)	blood	women undergoing lpsc due to urinary incontinence, pelvic organ prolapse, adnexal cysts
Walasik et al., 2023 [42]	Poland	2.5	24	25	no-no data	miR-3613-5p ↓, miR-451a ↑	plasma	women undergoing lpsc due to adnexal cysts or diagnostic lpsc
Chico-Sordo et al., 2024 [43]	Spain	5.6	77	82	yes, but only in control group-no effect	miR-30c-5p ↓	serum	women undergoing lpsc for sterilization or as oocyte donors
Liu et al., 2025 [44]	China	3.7	60	40	no-no data	miR-1229-5p ↑	endometriotic lesions	women undergoing operation for CIN III
Wang et al., 2025 [45]	China	2.3	100	80	no-no data	miR-375-3p ↓	serum	no operation-healthy women, with no symptoms and with normal levels of sex hormones

Note: ↑ indicates upregulated expression of miRNA; ↓ indicates downregulated expression of miRNA.

**Table 2:** Number of studies by continent.

Continent	Number of Studies
Asia	7 (China, Korea, Iran, Iraq)
Europe	6 (Spain, Austria, Belgium, Poland, Estonia)
North America	3 (USA)
Oceania	1 (Australia)

## Data Availability

The data that support the findings of this study are available on request from the corresponding author.
